# Oxybis(dimesitylborane) dichloro­methane hemisolvate

**DOI:** 10.1107/S160053680804275X

**Published:** 2009-02-06

**Authors:** Jung-Ho Son, James D. Hoefelmeyer

**Affiliations:** a Department of Chemistry, The University of South Dakota, 414 E. Clark St, Vermillion, SD 57069, USA

## Abstract

The title compound, C_36_H_44_B_2_O·0.5CH_2_Cl_2_, contains an almost linear O—B—O linkage [177.23 (15)°] and approximately orthogonal [interplanar angles 89.49 (5) and 80.77 (4)°] trigonal planar B centers, consistent with the previously reported non-solvated structure [Cardin*et al.* (1983). *J. Chem. Res. (S)*, p. 93]. Inter­molecular C—H⋯π inter­actions exist between mesityl groups, with a C—H⋯centroid separation of 3.6535 (18) Å. The dichloromethane mol­ecules lie on twofold rotation axes.

## Related literature

For the non-solvated structure, see: Cardin *et al.* (1983 ▶). For mol­ecular orbital calculations concerning the parent compound (H_2_B)_2_O, see: Fjeldberg *et al.* (1980 ▶).
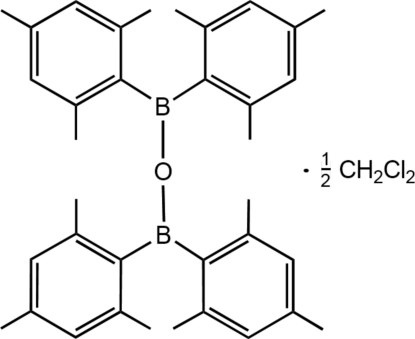


## Experimental

### 

#### Crystal data

C_36_H_44_B_2_O·0.5CH_2_Cl_2_
*M* *_r_* = 556.80
Monoclinic, 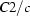

*a* = 36.563 (2) Å
*b* = 8.3129 (5) Å
*c* = 21.6346 (13) Å
β = 102.459 (1)°
*V* = 6420.9 (6) Å^3^
*Z* = 8
Mo *K*α radiation
μ = 0.15 mm^−1^
*T* = 100 (2) K
0.80 × 0.35 × 0.28 mm

#### Data collection

Bruker SMART APEXII diffractometer
Absorption correction: multi-scan (*SADABS*; Bruker, 2003 ▶) *T*_min_ = 0.892, *T*_max_ = 0.960
30958 measured reflections
5864 independent reflections
4475 reflections with *I* > 2σ(*I*)
*R*_int_ = 0.038

#### Refinement

*R*[*F* ^2^ > 2σ(*F* ^2^)] = 0.039
*wR*(*F* ^2^) = 0.108
*S* = 1.01
5864 reflections
378 parameters
H-atom parameters constrained
Δρ_max_ = 0.26 e Å^−3^
Δρ_min_ = −0.23 e Å^−3^

### 

Data collection: *SMART* (Bruker, 2003 ▶); cell refinement: *SAINT* (Bruker, 2003 ▶); data reduction: *SAINT*; program(s) used to solve structure: *SHELXTL* (Sheldrick, 2008 ▶); program(s) used to refine structure: *SHELXTL*; molecular graphics: *ORTEP-3* (Farrugia, 1997 ▶) and *PLATON* (Spek, 2003 ▶); software used to prepare material for publication: *SHELXTL*.

## Supplementary Material

Crystal structure: contains datablocks I, global. DOI: 10.1107/S160053680804275X/bi2327sup1.cif

Structure factors: contains datablocks I. DOI: 10.1107/S160053680804275X/bi2327Isup2.hkl

Additional supplementary materials:  crystallographic information; 3D view; checkCIF report

## Figures and Tables

**Table 1:** Hydrogen-bond geometry (Å, °)

*D*—H⋯*A*	*D*—H	H⋯*A*	*D*⋯*A*	*D*—H⋯*A*
C18—H18*A*⋯*Cg*^i^	0.98	2.80	3.649 (4)	145

Symmetry code: (i) 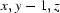. *Cg* is the centroid of the C19–C24 ring.

**Fig. 1.**
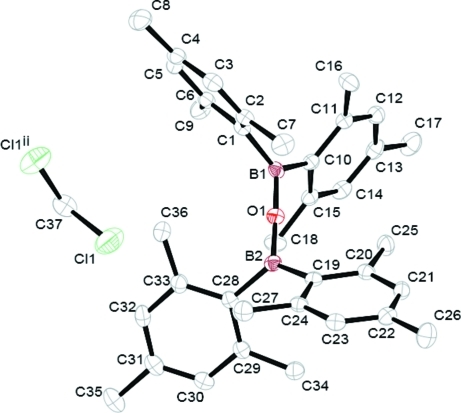
The molecular structure of the title compound with displacement ellipsoids drawn with 50% probability. H atoms are omitted. Symmetry code (ii): -x, y, -z + 1/2.

**Fig. 2.**
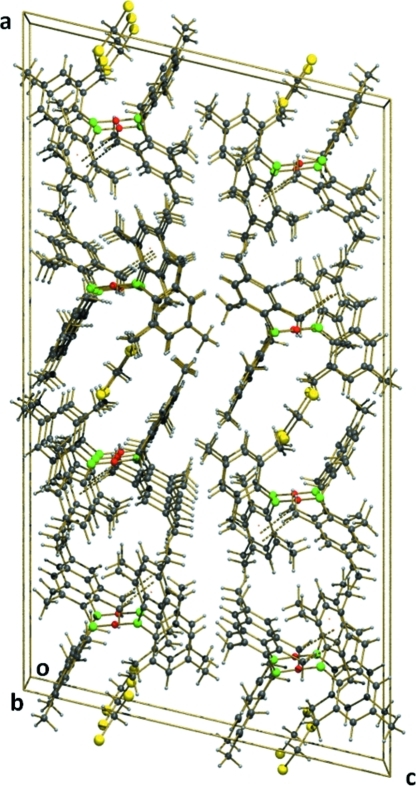
Packing diagram seen along the b axis. Dashed lines represent intermolecular C—H···π interactions.

## supplementary crystallographic information

### Comment

The overall geometry of the oxybis(dimesitylborane) molecule is very similar to the previously reported non-solvated structure (Cardin *et al.*, 1983). In the title compound, B—O = 1.351 (2) Å and B1—O1—B2 = 177.23 (15)°, compared with the non-solvated structure where B—O = 1.36 (2) Å and B—O—B = 165.5 (12) °. The angle between the boron trigonal planes (ψ) is 87.16 (5) ° which is larger than that of the previous structure (85 °). Ab *initio* molecular orbital calculation of (H_2_B)_2_O (Fjeldberg *et al.*, 1980) has predicted that as the B—O—B angle approaches linearity, the ψ angle should approach 90°, which is consistent with the present structure, showing B=O=B character. Orthogonality of the mesityl groups 89.49 (5) ° and 80.77 (4) ° attached on the same boron atom is a similar structural feature to the previous report (85.5 ° and 81.3 °; Cardin *et al.*, 1983). Intermolecular C—H···π interaction between mesityl groups exists with C···π separation of 3.6535 (18) ° (Table 1).

### Experimental

The title compound was isolated as a biproduct from the reaction of dimesitylboron fluoride and organolithium reagent in tetrahydrofuran. After removal of THF *in vacuo*, the residue was extracted with dichloromethane. The slow evaporation of dichloromethane under nitrogen atmosphere led to formation of colorless prismatic crystals. It is probable that unintended inclusion of water hydrolyzes the B—F bond to an intermediate borinic acid that undergoes condensation to form the B—O—B linkage.

### Refinement

H atoms were positioned geometrically with C—H (aromatic) = 0.95 Å and C—H (methyl) = 0.98 Å and allowed to ride on their parent atoms with *U*_iso_(H) = 1.2*U*_eq_(C) or *U*_iso_(H) = 1.5*U*_eq_(C), respectively.

### Crystal data

**Table:**

C_36_H_44_B_2_O·0.5CH_2_Cl_2_	*F*(000) = 2392
*M**_r_* = 556.80	*D*_x_ = 1.152 Mg m^−^^3^
Monoclinic, *C*2/*c*	Mo *K*α radiation, λ = 0.71073 Å
Hall symbol: -C 2yc	Cell parameters from 9936 reflections
*a* = 36.563 (2) Å	θ = 2.3–25.3°
*b* = 8.3129 (5) Å	µ = 0.15 mm^−^^1^
*c* = 21.6346 (13) Å	*T* = 100 K
β = 102.459 (1)°	Block, colourless
*V* = 6420.9 (6) Å^3^	0.80 × 0.35 × 0.28 mm
*Z* = 8	

### Data collection

**Table:**

Bruker SMART APEXII diffractometer	5864 independent reflections
Radiation source: fine-focus sealed tube	4475 reflections with *I* > 2σ(*I*)
graphite	*R*_int_ = 0.038
ω scans	θ_max_ = 25.4°, θ_min_ = 1.9°
Absorption correction: multi-scan (*SADABS*; Bruker, 2003)	*h* = −44→44
*T*_min_ = 0.892, *T*_max_ = 0.960	*k* = −10→10
30958 measured reflections	*l* = −26→26

### Refinement

**Table:**

Refinement on *F*^2^	Primary atom site location: structure-invariant direct methods
Least-squares matrix: full	Secondary atom site location: difference Fourier map
*R*[*F*^2^ > 2σ(*F*^2^)] = 0.039	Hydrogen site location: inferred from neighbouring sites
*wR*(*F*^2^) = 0.108	H-atom parameters constrained
*S* = 1.01	*w* = 1/[σ^2^(*F*_o_^2^) + (0.0493*P*)^2^ + 6.3252*P*] where *P* = (*F*_o_^2^ + 2*F*_c_^2^)/3
5864 reflections	(Δ/σ)_max_ = 0.001
378 parameters	Δρ_max_ = 0.26 e Å^−^^3^
0 restraints	Δρ_min_ = −0.23 e Å^−^^3^

### Special details

**Table:**

Geometry. All esds (except the esd in the dihedral angle between two l.s. planes) are estimated using the full covariance matrix. The cell esds are taken into account individually in the estimation of esds in distances, angles and torsion angles; correlations between esds in cell parameters are only used when they are defined by crystal symmetry. An approximate (isotropic) treatment of cell esds is used for estimating esds involving l.s. planes.
Refinement. Refinement of F^2^ against ALL reflections. The weighted R-factor wR and goodness of fit S are based on F^2^, conventional R-factors R are based on F, with F set to zero for negative F^2^. The threshold expression of F^2^ > 2sigma(F^2^) is used only for calculating R-factors(gt) etc. and is not relevant to the choice of reflections for refinement. R-factors based on F^2^ are statistically about twice as large as those based on F, and R- factors based on ALL data will be even larger.

### Fractional atomic coordinates and isotropic or equivalent isotropic displacement parameters (Å^2^)

**Table:**

	*x*	*y*	*z*	*U*_iso_*/*U*_eq_	Occ. (<1)
C1	0.07771 (4)	0.97659 (19)	0.14988 (7)	0.0191 (3)	
C2	0.07152 (5)	1.1445 (2)	0.14832 (7)	0.0220 (4)	
C3	0.03701 (5)	1.2062 (2)	0.11773 (8)	0.0253 (4)	
H3	0.0334	1.3195	0.1167	0.030*	
C4	0.00763 (5)	1.1077 (2)	0.08862 (8)	0.0257 (4)	
C5	0.01413 (5)	0.9432 (2)	0.08895 (8)	0.0246 (4)	
H5	−0.0053	0.8740	0.0681	0.030*	
C6	0.04829 (4)	0.8761 (2)	0.11892 (7)	0.0213 (4)	
C7	0.10174 (5)	1.2608 (2)	0.17920 (9)	0.0311 (4)	
H7A	0.1261	1.2224	0.1733	0.047*	
H7B	0.1021	1.2681	0.2245	0.047*	
H7C	0.0966	1.3671	0.1597	0.047*	
C8	−0.03016 (5)	1.1762 (3)	0.05847 (9)	0.0371 (5)	
H8A	−0.0270	1.2633	0.0296	0.056*	
H8B	−0.0422	1.2182	0.0915	0.056*	
H8C	−0.0458	1.0914	0.0348	0.056*	
C9	0.05242 (5)	0.6951 (2)	0.11911 (9)	0.0280 (4)	
H9A	0.0288	0.6463	0.0972	0.042*	
H9B	0.0588	0.6562	0.1629	0.042*	
H9C	0.0723	0.6654	0.0973	0.042*	
C10	0.14112 (4)	0.77985 (19)	0.16037 (7)	0.0197 (3)	
C11	0.15094 (4)	0.8088 (2)	0.10156 (8)	0.0219 (4)	
C12	0.17413 (5)	0.7014 (2)	0.07883 (8)	0.0248 (4)	
H12	0.1806	0.7232	0.0395	0.030*	
C13	0.18803 (4)	0.5636 (2)	0.11187 (8)	0.0252 (4)	
C14	0.17800 (5)	0.5345 (2)	0.16928 (8)	0.0245 (4)	
H14	0.1870	0.4398	0.1922	0.029*	
C15	0.15526 (4)	0.6395 (2)	0.19417 (8)	0.0212 (4)	
C16	0.13790 (5)	0.9584 (2)	0.06319 (8)	0.0288 (4)	
H16A	0.1427	1.0531	0.0907	0.043*	
H16B	0.1110	0.9501	0.0449	0.043*	
H16C	0.1516	0.9688	0.0291	0.043*	
C17	0.21420 (5)	0.4514 (2)	0.08774 (9)	0.0348 (5)	
H17A	0.2135	0.4750	0.0431	0.052*	
H17B	0.2064	0.3399	0.0919	0.052*	
H17C	0.2398	0.4665	0.1125	0.052*	
C18	0.14629 (5)	0.5979 (2)	0.25731 (8)	0.0272 (4)	
H18A	0.1512	0.4834	0.2662	0.041*	
H18B	0.1198	0.6210	0.2560	0.041*	
H18C	0.1620	0.6623	0.2907	0.041*	
C19	0.16666 (4)	1.13716 (19)	0.32859 (7)	0.0197 (3)	
C20	0.20008 (4)	1.1459 (2)	0.30583 (7)	0.0208 (4)	
C21	0.22615 (4)	1.2665 (2)	0.32754 (8)	0.0228 (4)	
H21	0.2486	1.2702	0.3121	0.027*	
C22	0.22038 (5)	1.3813 (2)	0.37104 (8)	0.0240 (4)	
C23	0.18715 (5)	1.3748 (2)	0.39217 (8)	0.0227 (4)	
H23	0.1825	1.4536	0.4213	0.027*	
C24	0.16034 (4)	1.25578 (19)	0.37186 (7)	0.0205 (4)	
C25	0.20941 (5)	1.0248 (2)	0.25940 (8)	0.0274 (4)	
H25A	0.1947	1.0484	0.2169	0.041*	
H25B	0.2035	0.9163	0.2719	0.041*	
H25C	0.2362	1.0314	0.2593	0.041*	
C26	0.24893 (5)	1.5117 (2)	0.39311 (10)	0.0342 (4)	
H26A	0.2432	1.6053	0.3651	0.051*	
H26B	0.2740	1.4715	0.3921	0.051*	
H26C	0.2482	1.5432	0.4365	0.051*	
C27	0.12447 (5)	1.2611 (2)	0.39576 (8)	0.0270 (4)	
H27A	0.1257	1.1820	0.4298	0.040*	
H27B	0.1032	1.2358	0.3610	0.040*	
H27C	0.1212	1.3689	0.4120	0.040*	
C28	0.12317 (4)	0.88673 (19)	0.36025 (7)	0.0199 (3)	
C29	0.14799 (5)	0.82528 (19)	0.41446 (7)	0.0212 (4)	
C30	0.13448 (5)	0.7269 (2)	0.45668 (8)	0.0236 (4)	
H30	0.1516	0.6857	0.4926	0.028*	
C31	0.09701 (5)	0.6873 (2)	0.44793 (8)	0.0258 (4)	
C32	0.07272 (5)	0.7474 (2)	0.39490 (8)	0.0261 (4)	
H32	0.0469	0.7201	0.3879	0.031*	
C33	0.08488 (5)	0.8468 (2)	0.35142 (8)	0.0238 (4)	
C34	0.18948 (5)	0.8623 (2)	0.42848 (8)	0.0266 (4)	
H34A	0.1994	0.8411	0.3907	0.040*	
H34B	0.2024	0.7940	0.4633	0.040*	
H34C	0.1934	0.9757	0.4405	0.040*	
C35	0.08310 (6)	0.5840 (2)	0.49550 (9)	0.0352 (5)	
H35A	0.0605	0.5263	0.4743	0.053*	
H35B	0.0773	0.6523	0.5290	0.053*	
H35C	0.1025	0.5062	0.5141	0.053*	
C36	0.05574 (5)	0.9111 (2)	0.29634 (8)	0.0328 (4)	
H36A	0.0317	0.9212	0.3090	0.049*	
H36B	0.0531	0.8367	0.2605	0.049*	
H36C	0.0636	1.0168	0.2839	0.049*	
C37	0.0000	0.3145 (3)	0.2500	0.0362 (6)	
H37A	0.0087	0.2445	0.2191	0.043*	0.50
H37B	−0.0087	0.2446	0.2809	0.043*	0.50
B1	0.11595 (5)	0.9032 (2)	0.18790 (8)	0.0190 (4)	
B2	0.13844 (5)	0.9922 (2)	0.31031 (9)	0.0200 (4)	
O1	0.12764 (3)	0.95073 (13)	0.24867 (5)	0.0210 (3)	
Cl1	0.037228 (16)	0.43420 (8)	0.28957 (3)	0.05884 (19)	

### Atomic displacement parameters (Å^2^)

**Table:**

	*U*^11^	*U*^22^	*U*^33^	*U*^12^	*U*^13^	*U*^23^
C1	0.0227 (8)	0.0190 (8)	0.0168 (8)	0.0005 (7)	0.0068 (6)	0.0006 (6)
C2	0.0266 (9)	0.0217 (9)	0.0188 (8)	0.0010 (7)	0.0074 (7)	0.0008 (7)
C3	0.0330 (10)	0.0213 (9)	0.0234 (9)	0.0060 (7)	0.0101 (7)	0.0024 (7)
C4	0.0247 (9)	0.0332 (10)	0.0196 (8)	0.0080 (8)	0.0062 (7)	0.0030 (7)
C5	0.0205 (8)	0.0311 (10)	0.0222 (9)	−0.0019 (7)	0.0046 (7)	−0.0015 (7)
C6	0.0236 (8)	0.0233 (9)	0.0180 (8)	−0.0005 (7)	0.0065 (7)	0.0002 (7)
C7	0.0363 (10)	0.0191 (9)	0.0361 (10)	−0.0011 (8)	0.0037 (8)	−0.0002 (8)
C8	0.0301 (10)	0.0460 (12)	0.0340 (10)	0.0148 (9)	0.0041 (8)	0.0011 (9)
C9	0.0274 (9)	0.0222 (9)	0.0321 (10)	−0.0024 (7)	0.0009 (7)	−0.0028 (8)
C10	0.0187 (8)	0.0184 (8)	0.0207 (8)	−0.0022 (6)	0.0013 (6)	−0.0024 (7)
C11	0.0202 (8)	0.0240 (9)	0.0205 (8)	−0.0010 (7)	0.0020 (7)	−0.0009 (7)
C12	0.0222 (9)	0.0293 (10)	0.0232 (9)	−0.0010 (7)	0.0060 (7)	−0.0039 (7)
C13	0.0194 (8)	0.0235 (9)	0.0307 (9)	0.0004 (7)	0.0013 (7)	−0.0082 (7)
C14	0.0232 (9)	0.0184 (9)	0.0290 (9)	0.0014 (7)	−0.0009 (7)	−0.0003 (7)
C15	0.0201 (8)	0.0191 (8)	0.0226 (8)	−0.0022 (7)	0.0006 (6)	−0.0008 (7)
C16	0.0336 (10)	0.0300 (10)	0.0248 (9)	0.0050 (8)	0.0108 (7)	0.0045 (8)
C17	0.0266 (9)	0.0358 (11)	0.0408 (11)	0.0071 (8)	0.0045 (8)	−0.0103 (9)
C18	0.0348 (10)	0.0209 (9)	0.0248 (9)	0.0025 (7)	0.0041 (7)	0.0034 (7)
C19	0.0221 (8)	0.0193 (8)	0.0166 (8)	0.0025 (7)	0.0018 (6)	0.0028 (6)
C20	0.0228 (8)	0.0204 (8)	0.0190 (8)	0.0021 (7)	0.0037 (7)	0.0023 (7)
C21	0.0194 (8)	0.0245 (9)	0.0249 (9)	0.0002 (7)	0.0055 (7)	0.0028 (7)
C22	0.0248 (9)	0.0192 (9)	0.0263 (9)	0.0006 (7)	0.0021 (7)	0.0019 (7)
C23	0.0284 (9)	0.0182 (8)	0.0209 (8)	0.0034 (7)	0.0038 (7)	−0.0002 (7)
C24	0.0227 (8)	0.0193 (8)	0.0186 (8)	0.0027 (7)	0.0027 (6)	0.0032 (7)
C25	0.0239 (9)	0.0320 (10)	0.0283 (9)	−0.0028 (8)	0.0100 (7)	−0.0069 (8)
C26	0.0311 (10)	0.0260 (10)	0.0460 (12)	−0.0059 (8)	0.0095 (8)	−0.0080 (9)
C27	0.0291 (9)	0.0249 (9)	0.0281 (9)	0.0007 (7)	0.0088 (7)	−0.0053 (7)
C28	0.0231 (8)	0.0180 (8)	0.0198 (8)	−0.0012 (7)	0.0069 (7)	−0.0038 (7)
C29	0.0245 (9)	0.0189 (8)	0.0215 (8)	0.0007 (7)	0.0080 (7)	−0.0037 (7)
C30	0.0315 (9)	0.0192 (9)	0.0209 (9)	0.0019 (7)	0.0072 (7)	−0.0014 (7)
C31	0.0353 (10)	0.0185 (9)	0.0273 (9)	−0.0007 (7)	0.0151 (8)	−0.0043 (7)
C32	0.0238 (9)	0.0261 (9)	0.0317 (10)	−0.0058 (7)	0.0135 (7)	−0.0077 (8)
C33	0.0230 (9)	0.0254 (9)	0.0242 (9)	−0.0016 (7)	0.0078 (7)	−0.0058 (7)
C34	0.0251 (9)	0.0279 (10)	0.0260 (9)	0.0012 (7)	0.0037 (7)	0.0046 (7)
C35	0.0462 (11)	0.0291 (10)	0.0364 (11)	−0.0050 (9)	0.0222 (9)	0.0006 (8)
C36	0.0211 (9)	0.0478 (12)	0.0299 (10)	−0.0019 (8)	0.0062 (7)	0.0009 (9)
C37	0.0462 (16)	0.0317 (15)	0.0323 (15)	0.000	0.0118 (12)	0.000
B1	0.0222 (9)	0.0163 (9)	0.0192 (9)	−0.0046 (7)	0.0063 (7)	0.0022 (7)
B2	0.0183 (9)	0.0219 (10)	0.0193 (9)	0.0042 (7)	0.0030 (7)	−0.0013 (7)
O1	0.0221 (6)	0.0214 (6)	0.0194 (6)	0.0000 (5)	0.0044 (5)	−0.0001 (5)
Cl1	0.0454 (3)	0.0613 (4)	0.0605 (4)	−0.0078 (3)	−0.0092 (3)	0.0073 (3)

### Geometric parameters (Å, °)

**Table:**

C1—C6	1.412 (2)	C20—C21	1.393 (2)
C1—C2	1.413 (2)	C20—C25	1.512 (2)
C1—B1	1.584 (2)	C21—C22	1.388 (2)
C2—C3	1.390 (2)	C21—H21	0.950
C2—C7	1.511 (2)	C22—C23	1.389 (2)
C3—C4	1.389 (2)	C22—C26	1.509 (2)
C3—H3	0.950	C23—C24	1.395 (2)
C4—C5	1.388 (2)	C23—H23	0.950
C4—C8	1.506 (2)	C24—C27	1.511 (2)
C5—C6	1.393 (2)	C25—H25A	0.980
C5—H5	0.950	C25—H25B	0.980
C6—C9	1.512 (2)	C25—H25C	0.980
C7—H7A	0.980	C26—H26A	0.980
C7—H7B	0.980	C26—H26B	0.980
C7—H7C	0.980	C26—H26C	0.980
C8—H8A	0.980	C27—H27A	0.980
C8—H8B	0.980	C27—H27B	0.980
C8—H8C	0.980	C27—H27C	0.980
C9—H9A	0.980	C28—C33	1.411 (2)
C9—H9B	0.980	C28—C29	1.414 (2)
C9—H9C	0.980	C28—B2	1.584 (2)
C10—C15	1.414 (2)	C29—C30	1.394 (2)
C10—C11	1.415 (2)	C29—C34	1.513 (2)
C10—B1	1.578 (2)	C30—C31	1.382 (2)
C11—C12	1.392 (2)	C30—H30	0.950
C11—C16	1.514 (2)	C31—C32	1.384 (2)
C12—C13	1.387 (2)	C31—C35	1.511 (2)
C12—H12	0.950	C32—C33	1.395 (2)
C13—C14	1.390 (2)	C32—H32	0.950
C13—C17	1.508 (2)	C33—C36	1.514 (2)
C14—C15	1.392 (2)	C34—H34A	0.980
C14—H14	0.950	C34—H34B	0.980
C15—C18	1.512 (2)	C34—H34C	0.980
C16—H16A	0.980	C35—H35A	0.980
C16—H16B	0.980	C35—H35B	0.980
C16—H16C	0.980	C35—H35C	0.980
C17—H17A	0.980	C36—H36A	0.980
C17—H17B	0.980	C36—H36B	0.980
C17—H17C	0.980	C36—H36C	0.980
C18—H18A	0.980	C37—Cl1^i^	1.7530 (17)
C18—H18B	0.980	C37—Cl1	1.7530 (17)
C18—H18C	0.980	C37—H37A	0.990
C19—C24	1.413 (2)	C37—H37B	0.990
C19—C20	1.414 (2)	B1—O1	1.351 (2)
C19—B2	1.580 (2)	B2—O1	1.351 (2)
			
C6—C1—C2	118.13 (15)	C22—C21—H21	119.0
C6—C1—B1	121.02 (14)	C20—C21—H21	119.0
C2—C1—B1	120.80 (14)	C21—C22—C23	117.89 (15)
C3—C2—C1	120.01 (16)	C21—C22—C26	120.84 (15)
C3—C2—C7	118.45 (15)	C23—C22—C26	121.25 (16)
C1—C2—C7	121.53 (15)	C22—C23—C24	121.97 (15)
C4—C3—C2	122.14 (16)	C22—C23—H23	119.0
C4—C3—H3	118.9	C24—C23—H23	119.0
C2—C3—H3	118.9	C23—C24—C19	119.96 (15)
C5—C4—C3	117.63 (15)	C23—C24—C27	118.31 (15)
C5—C4—C8	120.96 (17)	C19—C24—C27	121.71 (15)
C3—C4—C8	121.41 (17)	C20—C25—H25A	109.5
C4—C5—C6	122.14 (16)	C20—C25—H25B	109.5
C4—C5—H5	118.9	H25A—C25—H25B	109.5
C6—C5—H5	118.9	C20—C25—H25C	109.5
C5—C6—C1	119.91 (15)	H25A—C25—H25C	109.5
C5—C6—C9	118.57 (15)	H25B—C25—H25C	109.5
C1—C6—C9	121.48 (15)	C22—C26—H26A	109.5
C2—C7—H7A	109.5	C22—C26—H26B	109.5
C2—C7—H7B	109.5	H26A—C26—H26B	109.5
H7A—C7—H7B	109.5	C22—C26—H26C	109.5
C2—C7—H7C	109.5	H26A—C26—H26C	109.5
H7A—C7—H7C	109.5	H26B—C26—H26C	109.5
H7B—C7—H7C	109.5	C24—C27—H27A	109.5
C4—C8—H8A	109.5	C24—C27—H27B	109.5
C4—C8—H8B	109.5	H27A—C27—H27B	109.5
H8A—C8—H8B	109.5	C24—C27—H27C	109.5
C4—C8—H8C	109.5	H27A—C27—H27C	109.5
H8A—C8—H8C	109.5	H27B—C27—H27C	109.5
H8B—C8—H8C	109.5	C33—C28—C29	117.89 (15)
C6—C9—H9A	109.5	C33—C28—B2	121.55 (14)
C6—C9—H9B	109.5	C29—C28—B2	120.53 (14)
H9A—C9—H9B	109.5	C30—C29—C28	120.04 (15)
C6—C9—H9C	109.5	C30—C29—C34	118.02 (15)
H9A—C9—H9C	109.5	C28—C29—C34	121.94 (15)
H9B—C9—H9C	109.5	C31—C30—C29	122.08 (16)
C15—C10—C11	118.03 (15)	C31—C30—H30	119.0
C15—C10—B1	121.24 (14)	C29—C30—H30	119.0
C11—C10—B1	120.71 (14)	C30—C31—C32	117.89 (16)
C12—C11—C10	120.04 (15)	C30—C31—C35	120.77 (16)
C12—C11—C16	118.46 (15)	C32—C31—C35	121.33 (16)
C10—C11—C16	121.46 (15)	C31—C32—C33	122.14 (16)
C13—C12—C11	122.05 (16)	C31—C32—H32	118.9
C13—C12—H12	119.0	C33—C32—H32	118.9
C11—C12—H12	119.0	C32—C33—C28	119.95 (15)
C12—C13—C14	117.83 (16)	C32—C33—C36	117.84 (15)
C12—C13—C17	121.43 (16)	C28—C33—C36	122.20 (15)
C14—C13—C17	120.72 (16)	C29—C34—H34A	109.5
C13—C14—C15	122.07 (16)	C29—C34—H34B	109.5
C13—C14—H14	119.0	H34A—C34—H34B	109.5
C15—C14—H14	119.0	C29—C34—H34C	109.5
C14—C15—C10	119.97 (15)	H34A—C34—H34C	109.5
C14—C15—C18	118.05 (15)	H34B—C34—H34C	109.5
C10—C15—C18	121.97 (15)	C31—C35—H35A	109.5
C11—C16—H16A	109.5	C31—C35—H35B	109.5
C11—C16—H16B	109.5	H35A—C35—H35B	109.5
H16A—C16—H16B	109.5	C31—C35—H35C	109.5
C11—C16—H16C	109.5	H35A—C35—H35C	109.5
H16A—C16—H16C	109.5	H35B—C35—H35C	109.5
H16B—C16—H16C	109.5	C33—C36—H36A	109.5
C13—C17—H17A	109.5	C33—C36—H36B	109.5
C13—C17—H17B	109.5	H36A—C36—H36B	109.5
H17A—C17—H17B	109.5	C33—C36—H36C	109.5
C13—C17—H17C	109.5	H36A—C36—H36C	109.5
H17A—C17—H17C	109.5	H36B—C36—H36C	109.5
H17B—C17—H17C	109.5	Cl1^i^—C37—Cl1	110.83 (16)
C15—C18—H18A	109.5	Cl1^i^—C37—H37A	109.5
C15—C18—H18B	109.5	Cl1—C37—H37A	109.5
H18A—C18—H18B	109.5	Cl1^i^—C37—H37B	109.5
C15—C18—H18C	109.5	Cl1—C37—H37B	109.5
H18A—C18—H18C	109.5	H37A—C37—H37B	108.1
H18B—C18—H18C	109.5	O1—B1—C10	118.02 (14)
C24—C19—C20	118.17 (15)	O1—B1—C1	116.82 (14)
C24—C19—B2	120.44 (14)	C10—B1—C1	125.16 (14)
C20—C19—B2	121.24 (14)	O1—B2—C19	118.53 (15)
C21—C20—C19	119.94 (15)	O1—B2—C28	117.44 (15)
C21—C20—C25	117.82 (14)	C19—B2—C28	123.98 (14)
C19—C20—C25	122.22 (15)	B1—O1—B2	177.23 (15)
C22—C21—C20	122.04 (15)		
			
C6—C1—C2—C3	1.0 (2)	C21—C22—C23—C24	1.2 (2)
B1—C1—C2—C3	−176.35 (14)	C26—C22—C23—C24	179.48 (16)
C6—C1—C2—C7	−178.85 (15)	C22—C23—C24—C19	0.1 (2)
B1—C1—C2—C7	3.8 (2)	C22—C23—C24—C27	−178.11 (15)
C1—C2—C3—C4	0.7 (2)	C20—C19—C24—C23	−1.6 (2)
C7—C2—C3—C4	−179.45 (16)	B2—C19—C24—C23	174.05 (14)
C2—C3—C4—C5	−2.2 (2)	C20—C19—C24—C27	176.55 (15)
C2—C3—C4—C8	176.96 (16)	B2—C19—C24—C27	−7.8 (2)
C3—C4—C5—C6	2.1 (3)	C33—C28—C29—C30	0.8 (2)
C8—C4—C5—C6	−177.11 (16)	B2—C28—C29—C30	−177.25 (15)
C4—C5—C6—C1	−0.4 (2)	C33—C28—C29—C34	−179.47 (15)
C4—C5—C6—C9	177.65 (16)	B2—C28—C29—C34	2.4 (2)
C2—C1—C6—C5	−1.1 (2)	C28—C29—C30—C31	−0.6 (2)
B1—C1—C6—C5	176.20 (14)	C34—C29—C30—C31	179.65 (15)
C2—C1—C6—C9	−179.14 (15)	C29—C30—C31—C32	0.6 (2)
B1—C1—C6—C9	−1.8 (2)	C29—C30—C31—C35	−178.36 (16)
C15—C10—C11—C12	−0.6 (2)	C30—C31—C32—C33	−0.9 (3)
B1—C10—C11—C12	177.98 (15)	C35—C31—C32—C33	178.12 (16)
C15—C10—C11—C16	−178.30 (15)	C31—C32—C33—C28	1.1 (3)
B1—C10—C11—C16	0.2 (2)	C31—C32—C33—C36	−177.72 (16)
C10—C11—C12—C13	0.6 (2)	C29—C28—C33—C32	−1.1 (2)
C16—C11—C12—C13	178.41 (15)	B2—C28—C33—C32	177.00 (15)
C11—C12—C13—C14	0.1 (2)	C29—C28—C33—C36	177.72 (15)
C11—C12—C13—C17	−177.85 (16)	B2—C28—C33—C36	−4.2 (2)
C12—C13—C14—C15	−0.9 (2)	C15—C10—B1—O1	47.6 (2)
C17—C13—C14—C15	177.08 (15)	C11—C10—B1—O1	−130.88 (16)
C13—C14—C15—C10	1.0 (2)	C15—C10—B1—C1	−132.37 (16)
C13—C14—C15—C18	−179.35 (15)	C11—C10—B1—C1	49.1 (2)
C11—C10—C15—C14	−0.2 (2)	C6—C1—B1—O1	−126.16 (16)
B1—C10—C15—C14	−178.73 (15)	C2—C1—B1—O1	51.1 (2)
C11—C10—C15—C18	−179.88 (15)	C6—C1—B1—C10	53.8 (2)
B1—C10—C15—C18	1.6 (2)	C2—C1—B1—C10	−128.94 (17)
C24—C19—C20—C21	1.8 (2)	C24—C19—B2—O1	134.99 (16)
B2—C19—C20—C21	−173.80 (14)	C20—C19—B2—O1	−49.5 (2)
C24—C19—C20—C25	−179.95 (15)	C24—C19—B2—C28	−47.7 (2)
B2—C19—C20—C25	4.4 (2)	C20—C19—B2—C28	127.76 (17)
C19—C20—C21—C22	−0.5 (2)	C33—C28—B2—O1	−48.1 (2)
C25—C20—C21—C22	−178.86 (15)	C29—C28—B2—O1	129.90 (16)
C20—C21—C22—C23	−1.0 (2)	C33—C28—B2—C19	134.60 (17)
C20—C21—C22—C26	−179.27 (16)	C29—C28—B2—C19	−47.4 (2)

Symmetry codes: (i) −*x*, *y*, −*z*+1/2.

### Hydrogen-bond geometry (Å, °)

**Table:**

*D*—H···*A*	*D*—H	H···*A*	*D*···*A*	*D*—H···*A*
C18—H18A···Cg^ii^	0.98	2.80	3.649 (4)	145

Symmetry codes: (ii) *x*, *y*−1, *z*.
